# Cryptanalysis and improvement of an elliptic curve based signcryption scheme for firewalls

**DOI:** 10.1371/journal.pone.0208857

**Published:** 2018-12-13

**Authors:** Malik Zia, Rashid Ali

**Affiliations:** Department of Mathematics, Capital University of Science and Technology, Islamabad, Pakistan; King Saud University, SAUDI ARABIA

## Abstract

In network security, firewall is a security system that observes and controls the network traffic based on some predefined rules. A firewall sets up a barrier between internal network and another outside unsecured network, such as the Internet. A number of signcryption schemes for firewall are proposed over the years, many of them are proved to have security flaws. In this paper, an elliptic curve based signcryption scheme for firewalls is analyzed. It is observed that the scheme is not secure and has many security flaws. Anyone who knows the public parameters, can modify the message without the knowledge of sender and receiver. The claimed security attributes of non-repudiation, unforgeability, integrity and authentication are compromised. After successful cryptanalysis of this scheme, we proposed a modified version of the scheme.

## Introduction

**Fig 1:**
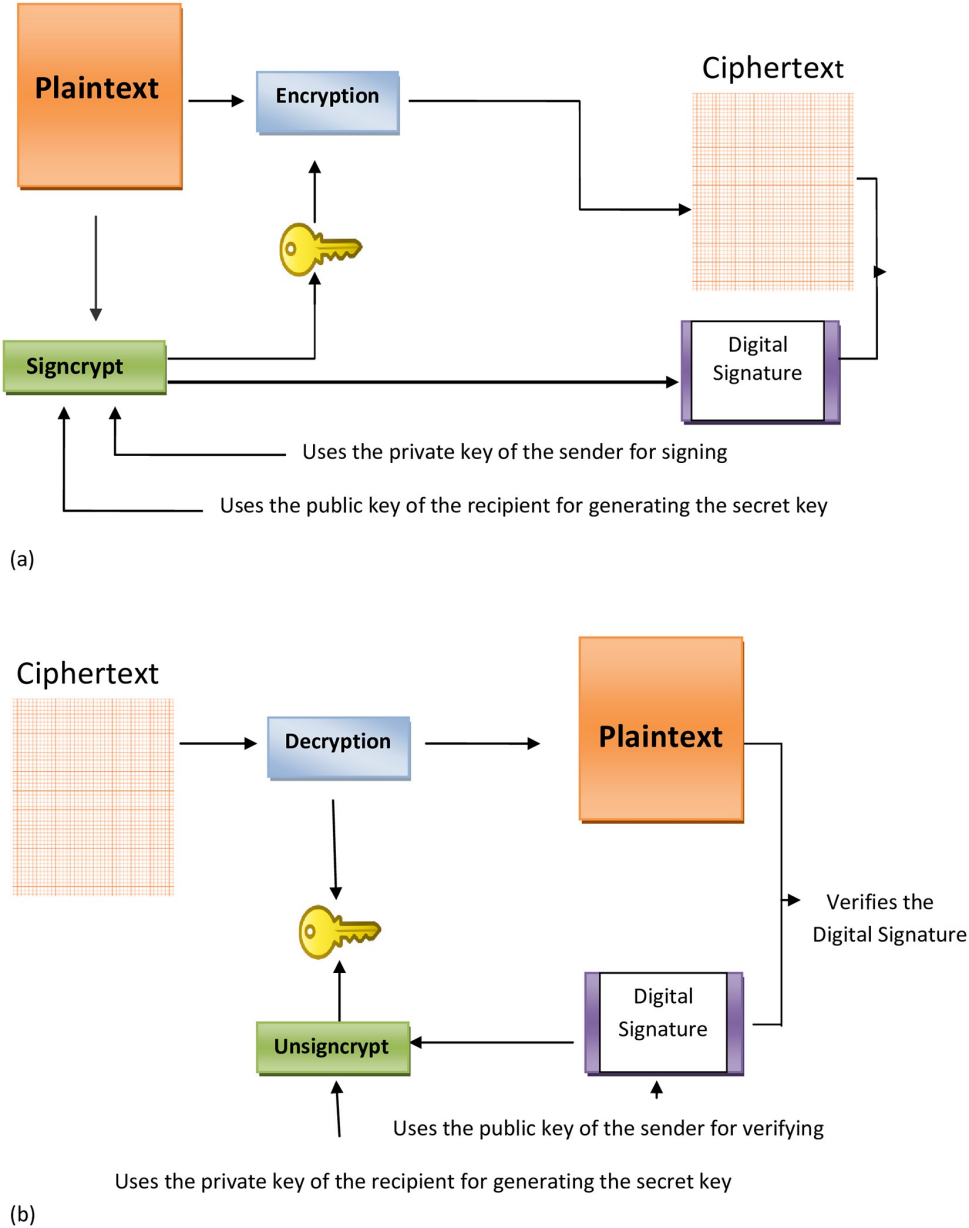
Signcryption model.

In 1997 *Zheng* [1] introduced a new cryptographic scheme named Signcryption, which fulfills the functionalities of digital signature and encryption in a single logical step as shown in Fig 1. In traditional public key cryptography the process of both data encryption and authentication is achieved by first digitally signing the document and then encrypting the signed document for transmission over a public network (i.e, signature-then- encryption). It has two drawbacks of low efficiency and high computational cost. A Signcryption scheme reduces the computational cost as compared to signature-then- encryption scheme.

Encryption and digital signature are two basic security properties of any singcryption scheme. Such properties include integrity, non-repudiation, unforgeability and confidentiality. Forward secrecy and public verifiability are additional features that are provided depending upon the requirements.

Various signcryption schemes were introduced over the years, each scheme having its own benefits and drawbacks. In *Zheng’s* signcryption scheme [1], the sender drives the secret key for symmetric encryption by using receivers public key. After receiving the signcrypted text, receiver gets the same secret key by using his private key. *Jung et al* [2] analysis shows that *Zheng* signcryption scheme [1] does not provide message confidentiality when the private key of sender is comporomised. He proposed a new signcryption scheme to overcome the drawbacks of *Zheng* [1] scheme with additional forward secrecy properity.

Later on, *Bao and Dang* [3] modified *Zheng’s* [1] scheme such that the judge can authenticate the signature without any use of recipient’s private key. Gamage et al [4] modified *Zheng* [1] scheme so that anyone can authenticate the signature of the corresponding ciphertext. The proposed is based upon discrete logarithm problem (DLP) for firewalls authentications but does not provide multi-reciever functionality. *Boneh et al* [5] proposed a new aggregate signature scheme which reduces the size of certificate chains. If there are *n* distinct messages and *n* distinct users then aggregating all distinct *n* signatures to a single short signature in such a way that each user assures the authenticity of received message. The proposed scheme reduces the computational and communication cost as compared to single signature schemes. *Horng et al* [6] proposed an efficient certificate less aggregate signature scheme for vehicular sensor networks. The proposed scheme achieves the conditional privacy preservation and it is secure against existential forgery on adaptively chosen plaintext attack. Their scheme has less computational overhead as compared to existing aggregate signature scheme. *Toorani* and *Shirazi* [7] introduced a signcryption scheme based on elliptic curve with additional forward secrecy property. *Selvi* [8] introduced an identity based signcryption technique for multiple receivers by using bilinear pairing. For some recent authentication protocols and their applications, we refer to the work presented in [9]–[12].

Firewall is a security system that monitors the network traffic based on some rules. Some schemes are suitable for firewalls but each has its own drawbacks and limitations.

Recently, Iqbal et al [13] introduced a new efficient signcryption scheme based on elleptic curve for firewalls. They claim that their scheme is secure and no one can duplicate the original message. In this paper, our analylsis shows that the scheme proposed in [13] is not secure and has many security flaws.

This paper is organized as: First we present the signcryption scheme introduced by Iqbal et al [13], followed by the cryptanalysis section. The improvement and modification of the scheme of the scheme is described in the next section. Later, the security analysis of the modified scheme is discussed, followed by the conclusion section.

## Signcryption scheme of Iqbal et al

Recently, Iqbal et al [13] proposed a new signcryption scheme for firewalls. The proposed scheme is based on elliptic curve cryptography. An elliptic curve over a finite field $F_{p}$ consists of the points satisfying the equation *y*^2^ = *x*^3^ + *ax* + *b* mod *p*, where *a*, *b* belongs to $F_{p}$ (the multiplicative group of integers mod *p*) along with a point *O* at infinity. The entire security of elliptic curve cryptography is based upon elliptic curve discrete logarithm problem that is, given points *A* and *B* = *nA* on an elliptic curve, it is computationally hard to find the integer *n*. For details on elliptic curve cryptography we refer to [14].

The basic aim of their proposed scheme is to present a new signcryption scheme for firewalls. The authors claim that proposed scheme provides security attributes of integrity, message confidentiality, signature unforgeability, public verifiability, non-repudiation, and forward secrecy properity. Their analysis shows that proposed scheme is computationally efficient as compared to alraedy existing signcryption schemes. The scheme proposed by Iqbal et al [13] is described below.

**Global parameters** Both Alice and Bob agreed on the following parameters (Table 1).

**Table 1:** Global parameters.

Variables	Description
p*	A large prime number, where p* > 2^1024^.
*E*_p*_(*a*, *b*)	An elliptic curve over *GF*(p*).
*G*	A base point *G* of a group of a very large order *q*.
*H*	A one way hash function.
*E* and *D*	Symmetric encryption and decryption algorithms.
*ID*_*i*_	Identifiers of sender and receiver from CA.

**Algorithm 1**. The Iqbal et al [13] scheme is described in four phases given below:

### 1. Key generation

- User A (Sender)
  - Selects an integer *n*_*A*_ randomly as a private key such that *n*_*A*_ < *q*
  - Calculates public key as elliptic curve point *P*_*A*_ = *n*_*A*_*G*
- User B (Receiver)
  - Selects an integer *n*_*B*_ randomly as a private key such that *n*_*B*_ < *q*
  - Calculate his Public key as elliptic curve point *P*_*B*_ = *n*_*B*_*G*

### 2. Signcryption

Suppose that Alice(sender) wants to transmit a message *m* over a public network to Bob(receiver). First Alice checks the Bob,s certificate and verifies his public key *P*_*B*_. She performs the following steps to send a signcrypted text.

1. Choose a random number *v* from [1, 2, 3….*q* − 1].
2. Calculate *R* = *vG* = (*x*_*R*_, *y*_*R*_).
3. Calculate *r* = (*v* + *n_A_*) mod *q*.
4. Calculate *Q* = *rP*_*B*_ = (*x*_*Q*_, *y*_*Q*_).
5. Calculate *k* = *H*(*x*_*Q*_||*ID*_*A*_||*y*_*Q*_||*ID*_*B*_).
6. Calculate ciphertext *C* = *E*_*k*_(*m*) by using symmetric encryption *E*_*k*_ with the secret key *k*.
7. Calculate *t* = *H*(*C*||*x*_*R*_||*ID*_*A*_||*y*_*R*_||*ID*_*B*_).
8. Calculate *s* = *rt*^−1^ mod *q*.
9. Sends (*C*, *R*, *s*) to reciever.

### 3. Signature verification by firewalls

This scheme enables firewalls to authenticate the signcrypted text (*C*, *R*, *s*) without reading the contents of the original message. Firewalls verify the signature of Alice by using signcrypted text (*C*, *R*, *s*). Only the ciphertext and public parameters are required to verify the signature unforgeability. Firewalls authentication consists of the following steps:

1. Receive (*C*, *R*, *s*) from the sender.
2. Calculate the elliptic curve point as P* = (*R* + *P*_*A*_).
3. Calculate *t* = *H*(*C*||*x*_*R*_||*ID*_*A*_||*y*_*R*_||*ID*_*B*_).
4. Firewalls authenticate the message *m* only if *stG* = P*.

### 4. Unsigncryption

1. Recieve (*C*, *R*, *s*) from sender.
2. Calculate the elleptic curve point P* = (*R* + *P*_*A*_).
3. Calculate *Q* = (*n*_*B*_)P* = (*x*_*Q*_, *y*_*Q*_).
4. Calculate *k* = *H*(*x*_*Q*_||*ID*_*A*_||*y*_*Q*_||*ID*_*B*_).
5. Find the plaintext *m* = *D*_*k*_(*C*) by using symmetric encryption scheme with shared key *k*.
6. Calculate *t* = *H*(*C*||*x*_*R*_||*ID*_*A*_||*y*_*R*_||*ID*_*B*_).
7. Accept the message *m* only if *stG* = P*.

## Cryptanalysis

**Fig 2:**
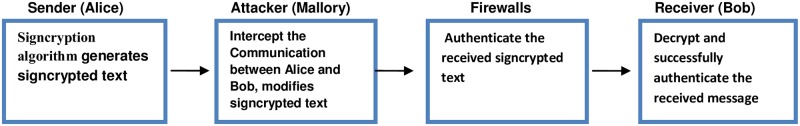
Our cryptoanalysis model.

In this section, Iqbal et al scheme [13] is cryptanalyzed. It is proved that the scheme has many security issues and weaknesses. Their scheme does not provide the message authenticity, unforgeability and non-repudiation. Mallory (an attacker) builds a new signcryption algorithm which generates the signcrypted text that is acceptable by unsigncryption algorithm. Suppose Mallory can intercept the network traffic between Alice and Bob and wants to generate a valid signcrypted text as described in Fig 2.

Mallory performs the following operations to transmit a message *m*′ of his choice.

### 1. Signcryption

1. Choose a random number *v*′ from [1, 2, 3….*q* − 1].
2. Calculate the elliptic curve point as $R^{\prime }=v^{\prime }G-P_{A}=\left(x_{R}^{\prime },y_{R}^{\prime }\right).$
3. Calculate the elliptic curve point as $Q^{\prime }=v^{\prime }P_{B}=\left(x_{Q}^{\prime },y_{Q}^{\prime }\right).$
4. Calculate the secret key as $k^{\prime }=H(x_{Q}^{\prime }||ID_{A}\left|\right|y_{Q}^{\prime }||ID_{B}).$
5. Calculate the ciphertext *C*′ = *E*_*k*_ ′(*m*′) by using symmetric encryption scheme *E*_*k*_ ′ with secret key *k*′.
6. Calculate the hash value as $t^{\prime }=H(C^{\prime }\left|\right|x_{R}^{\prime }||ID_{A}\left|\right|y_{R}^{\prime }||ID_{B}).$
7. The signature parameter *s*′ is calculated as $s^{\prime }={t^{\prime }}^{-1}v^{\prime }\;\mathrm{mod}\;q.$
8. Mallory sends (*C*′, *R*′, *s*′) to Bob.

### 2. Signature verification by firewalls

1. Receive (*C*′, *R*′, *s*′) from sender.
2. Calculate the elliptic curve point as P* = (*R*′ + *P*_*A*_).
3. Calculate $t^{\prime }=H(C^{\prime }\left|\right|x_{R}^{\prime }||ID_{A}\left|\right|y_{R}^{\prime }||ID_{B}).$
4. Firewalls authenticate the message *m*′ by verifying the relation *s*′*t*′*G* = P*.

### 3. Unsigncryption

1. Bob receives the text (*C*′, *R*′, *s*′).
2. Calculate the elliptic curve point as P* = (*R*′ + *P*_*A*_)
3. Calculate the elliptic curve point as $Q^{\prime }=\left(n_{B}\right)P^{*}=\left(x_{Q}^{\prime },y_{Q}^{\prime }\right)$
4. Calculate the secret key as $k^{\prime }=H(x_{Q}^{\prime }||ID_{A}\left|\right|y_{Q}^{\prime }||ID_{B}).$
5. Find the plaintext *m*′ = *D*_*k*′_(*C*′) by using symmetric encryption scheme with secret key *k*′.
6. Calculate the hash value as $t^{\prime }=H(C^{\prime }\left|\right|x_{R}^{\prime }||ID_{A}\left|\right|y_{R}^{\prime }||ID_{B}).$
7. Accept the message *m*′ by verifying *s*′*t*′*G* = P*.

In this way Mallory makes a fake signcrypted text of his choice and sends it to Bob. After receiving the signcrypted message (*C*′, *R*′, *s*′), first the firewalls successfully verifies the signature. Then at the receiver’s end unsigncryption algorithm verifies the signcrypted text and then decrypts the message. Bob now believes that the message is sending by authentic person Alice. In this way Mallory, defeats the cryptosystem and now able to send any signcrypted text of his own choice.

### 4. Correctness

The same secret key *k*′ is generated by Mallory and Bob. The elliptic curve point *Q*′, which is used for generation of secret key *k*′, is same.

$$\begin{array}{lll} Q^{\prime } & =\left(n_{B}\right)P^{*} & \\ & =\left(n_{B}\right)\left(R^{\prime }+P_{A}\right) & \left(\text{Step}\;(2)\;\text{Unsigncryption}\;\text{algorithm}\right)\\ & =\left(n_{B}\right)\left(v^{\prime }G-P_{A}+P_{A}\right) & \left(\text{Step}\;(2)\;\text{Signcryption}\;\text{algorithm}\right)\\ & =\left(n_{B}\right)\left(v^{\prime }G\right) & \\ & =v^{\prime }P_{B}=Q^{\prime } & \end{array}$$

After receiving the signcrypted text, unsigncryption algorithm correctly verifies the authenticity of received message.

$$\begin{array}{lll} s^{\prime }t^{\prime }G & =\left(t^{\prime -1}v^{\prime }\right)\left(t^{\prime }G\right) & \left(\text{Step}\;(7)\;\text{Signcryption}\;\text{algorithm}\right)\\ & =v^{\prime }G & \\ & =P^{*} & \end{array}$$

Moreover, this scheme has no protection against Man-At-The-End (MATE) attack. For details on MATE attack, we refer to the work of Akhunzada et al [15] and the references their in.

## Modification and improvement of Iqbal et al scheme

Our analysis shows that the claimed security properties of Iqbal et al [13] scheme are compromised. We modifies the scheme to ensure the basic properties of security. In proposed scheme, the method to generate common secret key is very weak. We modify the key generation process so that only authentic sender and receiver can generate valid common key. In step (5) of signcryption algorithm 1, we replace (*ID*_*A*_, *ID*_*B*_) to (*x*_*S*_, *y*_*S*_) in key generation phase. In our improved scheme, Only authentic sender can generate the signcrypted text that is verified by unsigncryption algorithm. In our improved scheme, global parameters and firwalls authentication is same as proposed by Iqbal et al [13].

**Algorithm 2**. Our modified signcryption scheme is described as:

### 1. Signcryption

1. Choose a random number *v* from [1, 2, 3….*q* − 1].
2. Calculate *R* = *vG* = (*x*_*R*_, *y*_*R*_).
3. Calculate *r* = (*v* + *n_A_*) mod *q*.
4. Calculate *Q* = *rP*_*B*_ = (*x*_*Q*_, *y*_*Q*_)
5. Calculate *S* = (*n*_*A*_)*P*_*B*_ = (*x*_*S*_, *y*_*S*_).
6. Calculate *k* = *H*(*x*_*Q*_||*x*_*S*_||*y*_*Q*_||*y*_*S*_).
7. Calculate the ciphertext *C* = *E*_*k*_(*m*) by using symmetric encryption *E*_*k*_ with secret key *k*.
8. Calculate *t* = *H*(*C*||*x*_*R*_||*ID*_*A*_||*y*_*R*_||*ID*_*B*_).
9. Calculate *s* = *t*^−1^*r* mod *q*.
10. Sends (*C*, *R*, *s*) to reciever.

### 2. Unigncryption

1. Recieve (*C*, *R*, *s*) from sender.
2. Calculate the elleptic curve point as P* = (*R* + *P*_*A*_).
3. Calculate *Q* = (*n*_*B*_)P* = (*x*_*Q*_, *y*_*Q*_).
4. Calculate *S* = (*n*_*B*_)*P*_*A*_ = (*x*_*S*_, *y*_*S*_).
5. Calculate *k* = *H*(*x*_*Q*_||*x*_*S*_||*y*_*Q*_||*y*_*S*_).
6. Find the plaintext message *m* = *E*_*k*_(*C*) by using symmetric encryption with secret key *k*.
7. Calculate *t* = *H*(*C*||*x*_*R*_||*ID*_*A*_||*y*_*R*_||*ID*_*B*_).
8. Accept the message *m* only if *stG* = P*.

### 3. Correctness

The same secret key *k* is generated by sender and receiver. The elliptic curve point *Q* is used for key generation, which is same in Step(4) of signcryption and Step(3) in Unsigncryption algorithm.

$$\begin{array}{lll} Q & =n_{B}P^{*}=n_{B}\left(R+P_{A}\right) & \left(\text{Step}\;(2)\;\text{unsigncryption}\;\text{algorithm}\right)\\ & =n_{B}\left(vG+n_{A}G\right) & \left(\text{Step}\;(\text{2})\;\text{Signcryption}\;\text{algorithm}\right)\\ & =\left(v+n_{A}\right)P_{B} & \left(\text{Key}\;\text{generation}\;\text{process}\right)\\ & =rP_{B}=Q & \left(\text{Step}\;(4)\;\text{Signcryption}\;\text{algorithm}\right) \end{array}$$

Receiver accept the message *m* only if the following equation is verified by unsigncryption algorithm.

$$\begin{array}{lll} stG & =\left(t^{-1}r\right)tG & \left(\text{Step}\;(\text{9})\;\text{Signcryption algorithm}\right)\\ & =rG=\left(v+n_{A}\right)G & \left(\text{Step}\;(\text{3})\;\text{Signcryption}\;\text{algorithm}\right)\\ & =vG+P_{A} & \left(\text{Key}\;\text{generation}\;\text{process}\right)\\ & =R+P_{A}=P^{*} & \left(\text{Step}\;(\text{2})\;\text{Unigncryption}\;\text{algorithm}\right) \end{array}$$

## Security analysis

The modified scheme provides the confidentiality of message. The common shared secret key *k* is used for symmetric encryption and decryption which is only known to sender and receiver. The scheme ensures authentication, as it is certificate based. The validity of certificates is verified in signcryption and unsigncryption phases. Bob (receiver) can verify that the received message is not altered by Mallory (attacker). So our scheme provides message integrity. Without the knowledge of private key *k* of Alice (sender), no one can generate the valid signcrypted text. Our scheme also provides signature unforgeability, non-repudiation, ciphertext-only authentication, public verification and forward secrecy of message confidentiality. The computational cost in signcryption, unsigncryption and signature verification phase is same as given in [13]. The communication cost of modified scheme is also same as in [13]. The comparison of modified scheme with the existing schemes is described in Table 2 below.

**Table 2:** Comparision of our modified scheme with existing schemes.

Signcryption Scheme	C	I	U	N	P	A	F	F.S
Zheng [1]	yes	yes	yes	yes	no	no	no	no
Gamage [4]	yes	yes	yes	yes	yes	yes	no	yes
Bao and deng [3]	yes	yes	yes	yes	no	no	no	no
Jung et al [2]	yes	yes	yes	yes	no	no	yes	no
Elkamchochi [16]	yes	yes	yes	yes	no	no	no	no
Zheng and Imai [17]	yes	yes	yes	yes	no	no	no	no
Mohamed [18]	yes	yes	yes	yes	yes	yes	no	yes
Han et al [19]	yes	yes	yes	yes	no	yes	no	no
Hwang e t al [20]	yes	yes	yes	yes	no	yes	no	no
Zhou [21]	yes	yes	yes	yes	no	yes	no	no
Iqbal et al [13]	yes	no	no	no	yes	no	yes	yes
Our Modified Scheme	yes	yes	yes	yes	yes	yes	yes	yes

C: Confidentiality, I: Integrity, U: Unforgebility, N: Non-repudiation, P: Public Verification, A: Authentication of ciphertext-only, F: Forward Secrecy, F.S: Firewall Suitability.

We now discuss some attack models for our improved signcryption scheme and give counter measures against these attacks.

### Man-At-The-End (MATE)attack

Previously Man-At-The-End (MATE) attack is neglected largely in security analysis by researchers because it is difficult to model, analyze and evaluate predominantly [15]. Since the attacker is human, therefore can utilize all the capabilities of a human mind. Beside the adversary has authorized and unlimited access to the device and this results in all security protections to stand up for an adversary for a specific period of time.

The MATE attack has different forms depending upon the physical scenario of compromised device. At an individual level, altering attack is possible in which adversary altered the integrity of piece of software [22]. In reverse engineering attack, the adversary trace the intellectual property rights from the device software and then disrupts the privacy right of vendor [23]. Similarly, in cloning attack an adversary creates and issues the copies of software by vilating the copyright laws [24]. Sometime an adversary may attavk by crafting his own exploit code using the publicly available codes to make it hard to be reconised by an antivirus software [25].

Although MATE attack is difficult to analyze and model but there are mechanism to protect your device. The techniques to protect against MATE attack are: digital asset protection, software protection, hardware protection and hardware -based software protection. For further details on core protection mechanism against this attack we refer to [15].

### Man-In-The middle-attack

In man-in-middle attack, an adversary intercepts the network traffic between two parties and alter the information in such a way that both parties believes they are communicating with each other. The proposed Signcryption scheme of Iqbal et al is not secure against man-in-middle attack and an active attacker modifies the signcrypted text that is verified by unsigncryption algorithm.

Our modified Signcryption scheme overcome this security issue and resist against man-in-middle attack. Adversary get the signcrypted text (*C*, *R*, *s*) from publicly transmitted message but unable to modify the signcrypted message of his choice. The private key of Alice is used for key generation process in Step(5) of signcryption algorithm and then used for signature generation in Step (9) of signcryption algorithm. If attacker generates a signcrypted text with any fake key then unsigncryption algorithm will not be able to verify the signature in Step (8) of unsigncryption algorithem and hence the message *M* will not be accepted.

## Conclusion

In this paper, the security of Iqbal et al [13] scheme is analyzed and it is proved that that it has many security flaws. In their proposed scheme, one can easily generate the signcrypted text of his choice that is acceptable by unsigncryption algorithm. Their scheme does not provide message authentication, integrity, non-repudiation and unforgeability as claimed in [13] (Table 1). We modified their scheme to ensure the compromised security attributes. Our improved scheme provides the security attributes of authentication, message confidentiality, unforgeability, integrity, non-repudiation, Public Verification, authentication of ciphertext-only, forward Secrecy and firewall Suitability. The comparison of the modified signcryption scheme with the existing schemes in the literature is highlighted in Table 2.
