# Correction: Cryptanalysis and improvement of an elliptic curve based signcryption scheme for firewalls

**DOI:** 10.1371/journal.pone.0271371

**Published:** 2022-07-08

**Authors:** Malik Zia Ullah Bashir, Rashid Ali

The first author’s name is spelled incorrectly. The correct name is: Malik Zia Ullah Bashir. The correct citation is: Zia Ullah Bashir M, Ali R (2018) Cryptanalysis and improvement of an elliptic curve based signcryption scheme for firewalls. PLoS ONE 13(12): e0208857. https://doi.org/10.1371/journal.pone.0208857.
